# Frailty in the Digital Age: Advancing Frailty Research With Wearable Technology

**DOI:** 10.1093/geroni/igaf122.640

**Published:** 2025-12-31

**Authors:** Jennifer Schrack, Amal Wanigatunga, Megan Huisingh-Scheetz

## Abstract

Phenotypic frailty is an established construct of health and function with aging. Generally assessed in laboratory and clinical settings, phenotypic frailty is defined using traditional measures of health and function, including self-reported low physical activity, exhaustion, and weight loss, and measured weakness and slowness. To date, there is limited evidence connecting phenotypic frailty with real-world markers of health and function. To this end, digital data from wearable sensors may provide unique insights into daily behaviors and their association(s) with frailty risk or recovery. This symposium will focus on 1) pooling frailty measures across large cohort studies to aid in quantifying and defining digital measures of physical activity by frailty status, 2) assessing how these digital physical activity measures are associated with frailty risk, and 3) understanding digital measures of interstitial glucose in prefrail older adults. Dr. Etzkorn will present methodology related to pooling frailty definitions among adults aged ≥70 years participating in BLSA, ARIC, ACHIEVE, NHATS, and STURDY. Dr. Schrack will present physical activity characteristics by frailty status in each of these studies and as overall normative data. Dr. Davoudi will present the association between features of accelerometry data from NHATS and risk of frailty at 1-year follow-up. Dr. Wanigatunga will present baseline continuous glucose monitoring data from the START sedentary behavior reduction trial. Dr. Huisingh-Scheetz will offer insights as discussant. Collectively, these presentations will provide novel insights into frailty status and risk using data from wearable digital sensors and highlight their utility for monitoring frailty-related outcomes in older adults.

